# Apert Syndrome: Review and report a case

**DOI:** 10.1016/S1808-8694(15)30621-2

**Published:** 2015-10-18

**Authors:** Gleicy V.S. Carneiro, Jener G. Farias, Fred A.P. Santos, Patrícia L. Lamberti

**Affiliations:** 1 Master's degree student in dentistry, dental surgeon, Universidade Federal da Bahia, UFBA.; 2 Adjunct professor of surgery in the dentistry course, Universidade Estadual de Feira de Santana, UEFS, and the Universidade Metropolitana de Educacao e Saude, UNIME.; 3 Dental surgeon.; 4 Adjunct professor of stomatology, Universidade Metropolitana de Educacao e Saude, UNIME. Universidade Estadual de Feira de Santana

**Keywords:** acrocephalosyndactyly, apert, syndrome

## INTRODUCTION

Apert's syndrome is an autosomal dominant inherited disease characterized mainly by achrocephalia due to synostosis of the coronary suture and by usually symmetrical syndactyly involving the four extremities.^1^, ^2^

In most cases the disorder results from a mutation in the father; its prevalence at birth is 1:65,0003 and the incidence in Asians is high.4 Mental retardation is common. The literature shows that this syndrome is caused by one of two mutations in the receptor 2 of the growth factor gene (FGFR2) involving two adjacent aminoacids.^3^

Treatment of these patients is done by multidisciplinary teams. Planning of surgery should be done in stages: craniotomy aims to decompress the brain and is done in infancy; advancement of the middle third improves airway-nasal flow, and may be done in puberty; and finally, orthognathic surgery improves occlusion and dental esthetics, and may be done in adolescence.

## CASE REPORT

DOS, a male black patient aged 19 years, a student who was the first child of three. The other two siblings were normal. His mother brought him to the Buccomaxillofacial Trauma Surgery Unit of the Santa Casa de Misericordia, Sao Felix, state of Bahia, with the following complaint: “I would like to have the appearance of my son improved.” The family history contained no report of similar cases. The mother said that the pregnancy for this patient was normal and had no type of physical, pharmacological or psychological trauma. He was the first child of a couple in their third decade of life.

**Figure 1:**
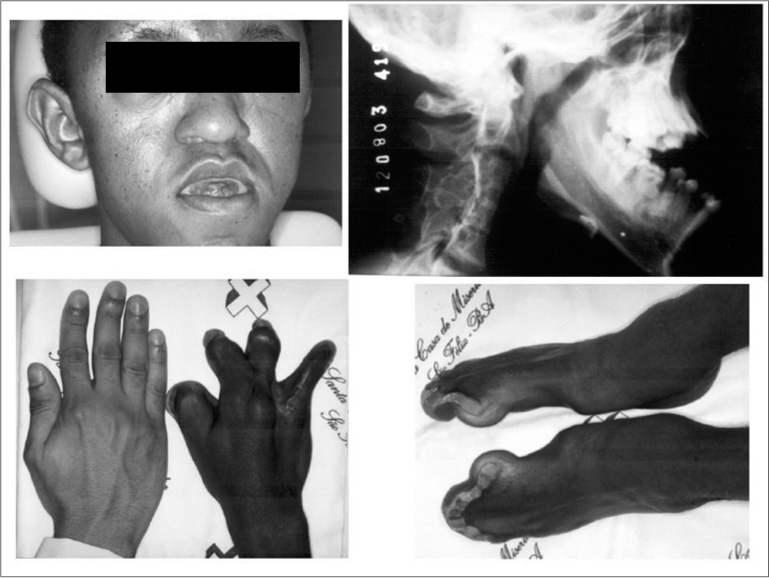
Medical and radiographic findings.

The physical examination showed the features of acrocephalosyndactyly (Figure 1). The face was mildly flattened and asymmetric, there was hypertelorism, ocular proptosis and depression of the lateral palpebral fissures. There was also a deep transversal groove above the supraorbital region, which gave the patient an aged look. The nose was small and its width was disproportional do its length; the nasal bridge was depressed, which gave it a “parrot's nose” aspect. The middle third of the face was hypoplasic, the nasolabial angle was decreased, and there was mouth breathing and absence of lip closure. The ears were wide and displaced downwards.

The fingers and toes were fused, at times forming a mass with a single nail.

The maxilla was atresic, the palatal ceiling was arched and there was gingival fibromatosis, which is typical of a pseudo-grooved palate. The teeth were generally ill positioned, with ectopic eruption, crowding and gyroversions. Toot eruption was delayed. Some teeth had been destroyed by caries, partially explained by lack of motor coordination. There was an anterior open bite and a posterior crossed bite.

A posteroanterior and lateral face X-ray and X-rays of the hands and feet were taken.

The lateral face X-ray shows clearly hypoplasia of the middle third and mandibular pseudoprognathism, demonstrating the bone discrepancy and decreased anteroposterior size of the cranium, as well as increased vertical length, yielding a turricephalic aspect. Hand and feet radiographs showed the pathognomonic syndactyly of Apert's syndrome.

The patient underwent nine surgical procedures to separate the hand fingers so that he could learn to write and carry out basic care such as holding objects and food and personal hygiene. Surgery was successful and the patient is currently independent in some tasks.

He was eventually referred to the Facial Deformity Unit of the Santo Antonio Hospital, Salvador, Bahia, where he is undergoing preoperative testing. He continues to be monitored at the unit in which the diagnosis was made.

## FINAL COMMENTS

In these heterogeneous dysplasias, each case has to be classified individually. The aim is to alert clinicians and surgeons about the prognosis and possible correction - including esthetic ones - for increased acceptance by the patient. The importance of social adjustment and the need for occupational therapy and physical activities should not be underestimated. The benefits are both emotional and physical, and postpone major malformations caused by synostosis.
